# An atlas of *Brachypodium distachyon* lateral root development

**DOI:** 10.1242/bio.060531

**Published:** 2024-09-02

**Authors:** Cristovāo de Jesus Vieira Teixeira, Kevin Bellande, Alja van der Schuren, Devin O'Connor, Christian S. Hardtke, Joop E. M Vermeer

**Affiliations:** ^1^ Laboratory of Molecular and Cell Biology, Institute of Biology, University of Neuchâtel, 2000 Neuchâtel, Switzerland; ^2^ IPSiM, University of Montpellier, CNRS, INRAE, Institut Agro, 34060 Montpellier, France; ^3^ Department of Plant Molecular Biology, University of Lausanne, 1015 Lausanne, Switzerland; ^4^ Sainsbury Lab, University of Cambridge, CB2 1LR Cambridge, UK

**Keywords:** *Brachypodium distachyon*, Lateral roots, Endodermis, Exodermis, Organogenesis

## Abstract

The root system of plants is a vital part for successful development and adaptation to different soil types and environments. A major determinant of the shape of a plant root system is the formation of lateral roots, allowing for expansion of the root system. *Arabidopsis thaliana*, with its simple root anatomy, has been extensively studied to reveal the genetic program underlying root branching. However, to get a more general understanding of lateral root development, comparative studies in species with a more complex root anatomy are required. Here, by combining optimized clearing methods and histology, we describe an atlas of lateral root development in *Brachypodium distachyon*, a wild, temperate grass species. We show that lateral roots initiate from enlarged phloem pole pericycle cells and that the overlying endodermis reactivates its cell cycle and eventually forms the root cap. In addition, auxin signaling reported by the DR5 reporter was not detected in the phloem pole pericycle cells or young primordia. In contrast, auxin signaling was activated in the overlying cortical cell layers, including the exodermis. Thus, Brachypodium is a valuable model to investigate how signaling pathways and cellular responses have been repurposed to facilitate lateral root organogenesis.

## Teaser

**Summary:** This study provides an atlas of lateral root development of the model grass species Brachypodium distachyon.

## INTRODUCTION

Root branching is vital for plant survival as it facilitates the uptake of water and nutrients (Orosa-Puente et al., 2018). Root system architecture (RSA) consists of structural features like root length, spread, number, and length of lateral roots (LRs), among others (Bao et al., 2014; Morris et al., 2017; Kumar et al., 2019). RSA exhibits great plasticity in response to environmental changes and it is a desirable trait to breed more resilient crops (Ye et al., 2017; Yu et al., 2019; Schäfer et al., 2022). In both monocots and dicots, the growth angle and number of LRs are the central components of the overall RSA (Atkinson et al., 2014; Roychoudhry et al., 2017). However, the molecular and cell biological programs underlying root branching are less described for major crops due to the difficulty of observing the root system throughout the plant's life cycle (Hochholdinger and Zimmermann, 2008).

Due to the relatively simple organization of its root system, tissue transparency and extensive genetic toolbox, *Arabidopsis thaliana* (Arabidopsis) has been the most characterized experimental system for dissecting the molecular mechanisms underlying LR development (Banda et al., 2019). In Arabidopsis, LRs initiate from lateral root founder cells (LRFCs), and a series of highly coordinated cell divisions leads to the development of a new LR primordium (LRP) (Casimiro et al., 2001; Ditengou et al., 2008; Stoeckle et al., 2018; Gala et al., 2021). In this case, LRFCs are patterned along the primary root axis with a regulated spacing, starting from the basal root meristem (Lavenus et al., 2015; Chen et al., 2018; Kircher and Schopfer, 2018; Torres-Martínez et al., 2020). Early stage LRP are more likely to initiate closer to the root tip. The first morphological event of LR initiation takes place in the differentiation zone where LRFC founders cells divide asymmetrically and anticlinal (Malamy and Benfey, 1997). In addition, auxin signaling in the neighboring endodermis plays a major role during LRP formation as blocking auxin responses in this tissue abolishes LR formation (Vermeer, et al., 2014). Subsequent periclinal and anticlinal divisions give rise to an organized dome shaped LRP (Malamy and Benfey, 1997).

In monocots, LR studies have mostly been conducted on rice and maize (Wang et al., 2002; Hochholdinger and Zimmermann, 2008; Jansen et al., 2012; Uga et al., 2013). Notably, LR initiation in monocots predominantly occurs in the phloem-associated pericycle (Jansen et al., 2013; Hardtke and Pacheco-Villalobos, 2015) and the underlying mechanisms governing the patterning of LR formation in these agriculturally important crops are not well described. In contrast to Arabidopsis, in monocots and many other plant species, during LR development both the pericycle and endodermis undergo cell divisions, thereby contributing to the formation of the new organ (Casero et al., 1995; Rebouillat et al., 2009; Banda et al., 2019; Xiao et al., 2019). However, only few studies have investigated the auxin-mediated transcriptome changes underlying LRP formation in monocots (Stelpflug et al., 2016; Kortz et al., 2019). Moreover, it is still unknown which signal is regulating the cell divisions in the endodermis overlying the newly formed LR.

The endodermis is the innermost cortical cell layer surrounding the vasculature (Geldner, 2013). Casparian strips (CS) and suberin lamellae (SL) formed in this layer were shown to be crucial in regulating the uptake of nutrients, in the response to osmotic stress and protection against pathogens (Ranathunge et al., 2008; Barberon et al., 2016). Additionally, a wide range of plant species have an additional apoplastic diffusion barrier localized just beneath the epidermis, known as the hypodermis or exodermis (Enstone et al., 2002). The term exodermis is used when the hypodermis contains a localized lignification and suberin deposition in its cell walls, serving a similar function as a barrier as the endodermis (Enstone et al., 2002; Kajala et al., 2021; Manzano et al., 2022 preprint). The exodermis differs from the endodermis in its pattern of differentiation. In maize roots for instance, the CS follows a synchronous development pattern, as ring-like structures, within the entire endodermis. In later stages of development, the CS increase in width, thereby enclosing the entire central cylinder (Enstone et al., 2002). The SLs are deposited later, but less synchronously, starting from a patchy zone that will develop in a fully suberized endodermis (with exception from the passage cells), depending on the growth conditions (Enstone et al., 2002; Kreszies et al., 2020; Andersen et al., 2021; Sexauer et al., 2021). In contrast, the development of the exodermis in maize is rather irregular in both radial and longitudinal directions (Líška et al., 2016). A recent study has also shown that suberization in the exodermis is essential for survival of tomato under drought conditions, revealing an important physiological function for this cell type (Cantó-Pastor et al., 2024). However, we still lack insights on how these two layers are involved in the emergence of the LRP.

Using crop plants for conducting LR studies is a challenging task due to their demanding growth requirements (Garvin, 2007; Scholthof et al., 2018). Instead, the wild grass *Brachypodium distachyon* (Brachypodium), possesses several characteristics that make it an excellent monocot model for studying LR development (Raissig and Woods, 2022). Brachypodium has a relatively small genome size, simple growth requirements, fast regeneration time, and exhibits self-pollination. Its embryonic root anatomy consists of a single axial primary root with seminal and leaf node roots developing later depending on the growth conditions. The general radial organization of the primary root consists of an epidermis, five cortex layers, and a single endodermis (Hardtke and Pacheco-Villalobos, 2015). The stele is surrounded by a single pericycle, and the vasculature is arranged with alternating xylem and phloem poles. Most of the above features are closely similar to root anatomy described in major cereal crops (Chochois et al., 2012; Hardtke and Pacheco-Villalobos, 2015; Raissig and Woods, 2022) including the site of LR initiation (Yu et al., 2016). For instance, in rice, maize and barley LRs initiate from cell divisions in pericycle cells associated to the protophloem, so-called phloem pole pericycle cells (Jansen et al., 2013; Ni et al., 2014; Xiao et al., 2019). Thus, the Brachypodium root system exhibits a high degree of developmental and anatomical similarity to important cereal crops, but with less complexity.

In this study, we present a developmental atlas describing the developmental stages of LR development in Brachypodium. We show that the endodermis reactivates its cell cycle and appears to contribute to the formation of the root cap and the columella cells of the emerged LRs. Furthermore, our results indicate the auxin signaling, as reported by DR5 promoter activity, is not evident in the phloem pole pericycle and during the early stages of LR development. Instead, auxin responses rather appear to be correlated with cell wall modifications during the emergence of the LRP. We show that early suberin deposition in roots appear to be controlled by water and nutrient availability and LRPs emerge towards the growth medium. Finally, we propose that the observed lignification pattern in the exodermis suggests a possible role in contributing to LRP emergence.

## RESULTS

### LRs initiate from phloem pole pericycle cells in Brachypodium

**Fig. 1.**
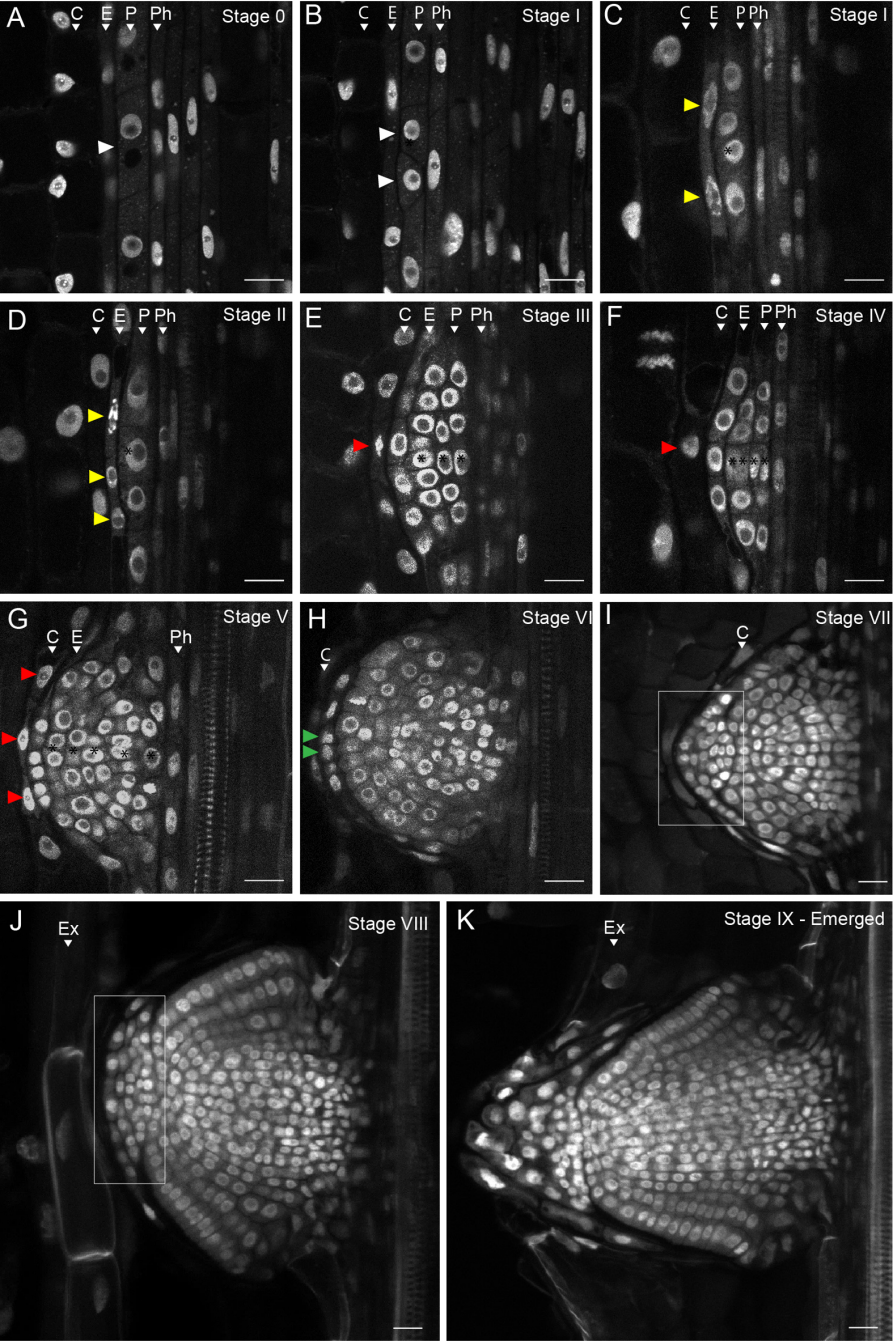
**Different stages of LRP development in Brachypodium.** (A) Stage 0: No discernible cell divisions in the pericycle cells. (B) Stage I: White arrowheads indicate the first anticlinal cell division in the pericycle. (C) Stage I: Yellow arrowheads indicate the flattening of the endodermal cells preceding the cell divisions in the next stage. (D) Stage II: The endodermis starts to divide anticlinal (yellow arrowheads). (E) Stage III: Periclinal divisions take place at the center of the LRP resulting in three layers of cells. The red arrowhead indicates cell divisions in the overlying cortex. (F) Stage IV: The LRP undergoes radial expansion through constant anticlinal and periclinal cell divisions in the center of the LRP. Four cell layers can be observed. (G) Stage V: Five to six cell layers can still be distinguished. LRP boundaries are established, and the endodermis appears to become integrated in the LRP. Red arrowheads indicate more cell divisions in the cortex layer in the vicinity of the LRP. (H) Stage VI: The endodermal cells on the apex of the LRP start to divide again (green arrowheads). Cell layer counting is no longer used from this stage. (I) Stage VII: Formation of the root cap (white rectangular area. (J) Stage VIII: The LRP reaches the root exodermis. (K) Stage IX: Emerged: The LRP is fully formed and traverses the exodermis and epidermis. Ex, exodermis; C, cortex; E, endodermis; P, pericycle; Ph, phloem. Representative images were obtained from 45 seedling roots from three independent replicates each consisting of at least 15 plants of Bd21-3. Samples were cleared with DEEP-Clear and stained with 0.01% propidium iodide. Scale bars: 20 μm.

To characterize the sequential developmental stages during LR development in the Brachypodium accession Bd21-3, we adapted the DEEP-CLEAR (Pende et al., 2020) protocol for plant tissue to clear roots and used propidium iodide (PI) to visualize the nuclei of the cleared roots via multiphoton microscopy (Fig. 1, Fig. S1). To categorize the LRP development in Brachypodium, we used the model described for Arabidopsis (Malamy and Benfey, 1997) with a few adaptations in the later developmental stages:

Stage I: Cell divisions occurring in pericycle cells adjacent to the phloem poles (between two xylem poles) are the first anatomical signs of LR initiation (Fig. 1B, C, Fig. S1B).

Stage II: This stage is marked by the initiation of the first anticlinal cell divisions in the endodermal cells overlying a Stage I LRP. Subsequently, the cells within the LRP undergo periclinal divisions while endodermal cells continue to divide anticlinal (Fig. 1D, Fig. S1C).

Stage III: Periclinal divisions occur in the center of the LRP resulting in the formation of three layers of cells. In parallel, the endodermis continues to divide anticlinal forming a boundary that spans the entire LRP. Additionally, the innermost cortex cell layer, in contact with the endodermis, appears to flatten (Fig. 1E, Fig. S1D).

Stage IV: This stage is characterized by the ongoing radial expansion of the LRP through additional anticlinal and periclinal divisions in its central region (Fig. 1F). As a result, the canonical dome shape becomes apparent, and four layers of cells can be counted. Furthermore, ongoing anticlinal cell divisions in the first cortical cell layer were observed, although these cells did not appear to become incorporated in the LRP (Fig. 1F).

Stage V: Five distinct cortical cell layers remain discernible. The LRP boundaries become well-defined, and the cell divisions patterns indicate that the endodermis-derived cells are integrated within the LRP (Fig. 1G, Fig. S1E).

Stage VI: In this stage the first periclinal endodermal cell divisions occur at the apex of the LRP suggesting the initiation of the lateral root cap formation (Fig. 1H, Fig. S1E). On average, LRP still have six to seven layers of cells. However, due to the increasing number of cell divisions in the central part of the LRP, it is impossible to apply the cell layer counting system for the remaining LRP developmental stages.

Stage VII: The LRP resembles a mature root tip containing an early developmental stage of the lateral root cap that continues to divide anticlinal (Fig. 1I, Fig. S1F).

Stage VIII: At this stage the LRP reaches the exodermis.

Stage IX: The LRP crosses the exodermis and epidermis characterizing its full emergence towards the root surface. Fig. S2 shows an illustration for the LRP developmental stages in Brachypodium.

### DR5pro::ER-mRFP does not detect transcriptional auxin responses during early stages of LR development

**Fig. 2.**
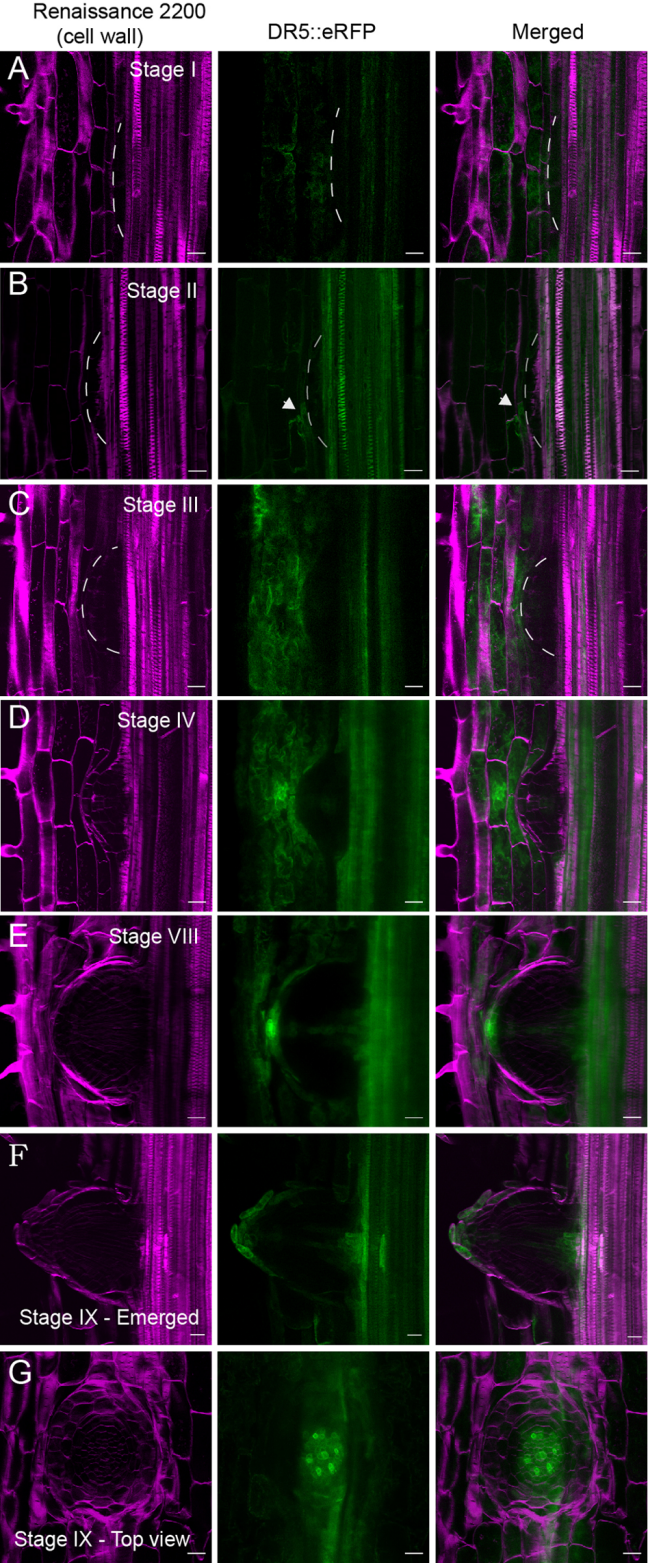
**DR5pro::ER-mRFP activity during LR development in Brachypodium.** (A) The DR5 signal is not evident in Stage I during the first pericycle cell divisions. (B) The DR5 signal could be observed in Stage II when the endodermis starts to divide (white arrowheads). (C,D) The DR5 signal is no longer observed in the endodermis but in the cortex cell layer in the vicinity of the LRP and in the central part of the LRP resembling vasculature. (E) The DR5 signal is intensified at the apex of the LRP, in the vasculature, and in the last cortex cell layer. (F,G) A fully emerged LR shows a similar DR5 pattern as usually observed in the primary root. Representative images were obtained from 45 seedlings from three independent replicates each consisting of at least 15 plants of Bd21-3. *DR5pro::ER-mRFP* (green) and cell walls stained with SCRI Renaissance (magenta) for cellulose. Scale bars: 50 μm.

The phytohormone auxin plays a crucial role during all stages of LRP development in many plant species including important cereal crops such as rice (Lin and Sauter, 2019), maize (Yu et al., 2019) and barley (Kirschner et al., 2017). However, most of the insights on how auxin signaling coordinates LR development comes from studies in Arabidopsis (Fukaki and Tasaka, 2009; Vermeer et al., 2014; Guseman et al., 2015; Cavallari et al., 2021), and much less is known whether discrete auxin-driven developmental modules have a similar role in monocots. To characterize transcriptional responses to auxin during LR development, we utilized a *DR5pro::ER-mRFP* marker line (van der Schuren et al., 2018). In contrast to what was described for LR initiation in maize (Jansen et al., 2013) we were unable to observe a clear DR5pro::ER-mRFP signal in phloem pole pericycle cells and in Stages I-II LRP (Fig. 2A,B); thus, making it challenging to correlate tissue specific changes in auxin responses with LRFC specification and LR initiation. We did observe a weak DR5pro::ER-mRFP signal in the endodermis and most inner cortical cells overlying Stage I-II LRP (Fig. 2A,B). The earliest detectable DR5pro::ER-mRFP signal in the LRP was only observed at Stage III, when the endodermis is already actively dividing (Fig. 2C). In later stages, the DR5pro::ER-mRFP signal was no longer detected in the endodermis but it gradually intensified at the apex of the growing LRP (Fig. 2D). As the LRP developed (Stages IV to VIII), we observed an increased DR5pro::ER-mRFP signal in the cortical cell layers overlying the developing LRP (Fig. 2D,E). Prior to and after emergence, the DR5pro::ER-mRFP signal in the newly-formed LR exhibited an expression pattern comparable to the main root tip (Fig. 2E-G and Fig. S4). Notably, we also observed a clear DR5pro::ER-mRFP signal in the exodermis cells overlying the LRP (Fig. S4). Although we have observed that LR development is induced by auxin treatment in Brachypodium (Fig. S6), we failed to detect a DR5pro::ER-mRFP signal in Stage I LRP. Based on these observations, we characterized whether SISTER of PIN-FORMED 1 (SoPIN1) and AUXIN RESISTANT 1 (AUX1), known transporters involved in auxin efflux and import, respectively, were expressed in early stage LRP (Marchant et al., 2002; Reinhardt et al., 2003; O'Connor et al., 2017). The presence of SoPIN1-Citrine was evident as early as Stage I (Fig. S5A). Later, signal was observed in the endodermis coinciding with its initial cell divisions in the endodermis Stage II (Fig. S5A). Subsequently, in later stages, SoPIN1-Citrine expression became predominantly localized in the central region of the LRP (Fig. S5A). Conversely, AUX1-sGFP exhibited expression within the LRP starting from Stage I initially in the phloem-pole pericycle and in the flanking regions with its intensity increasing subsequently in both the vasculature and endodermis (Stages III to V). Robust expression within the vasculature was also observed in Stages V and VI (Fig. S5B).

### Endodermal-derived cells appear to form the root cap of LRs

Next, we investigated whether the endodermal cells that reactivated their cell cycle and underwent anticlinal and periclinal divisions contribute to the formation of the columella of the LRP. Starch granule formation serves as a marker for differentiation of the columella cells (Guyomarc'h et al., 2012; Roychoudhry et al., 2023). To assess columella formation, we utilized Lugol's staining in conjunction with our histological clearing approach. Columella cells (boxed area in Fig. 5) were characterized by the presence of sediments of amyloplasts. Even though the endodermis starts to undergo periclinal divisions from Stage V (Fig. 5B,C), starch accumulation was only observed in the first cell layer of the columella during late Stage VI-VII (Fig. 5E,F), following numerous rounds of periclinal cell divisions. The intensity of Lugol's staining gradually intensified from Stage VII to the fully emerged LRP (Fig. 5H).

### Suberin deposition in the exodermis of Brachypodium roots is delayed compared to the endodermis

**Fig. 3.**
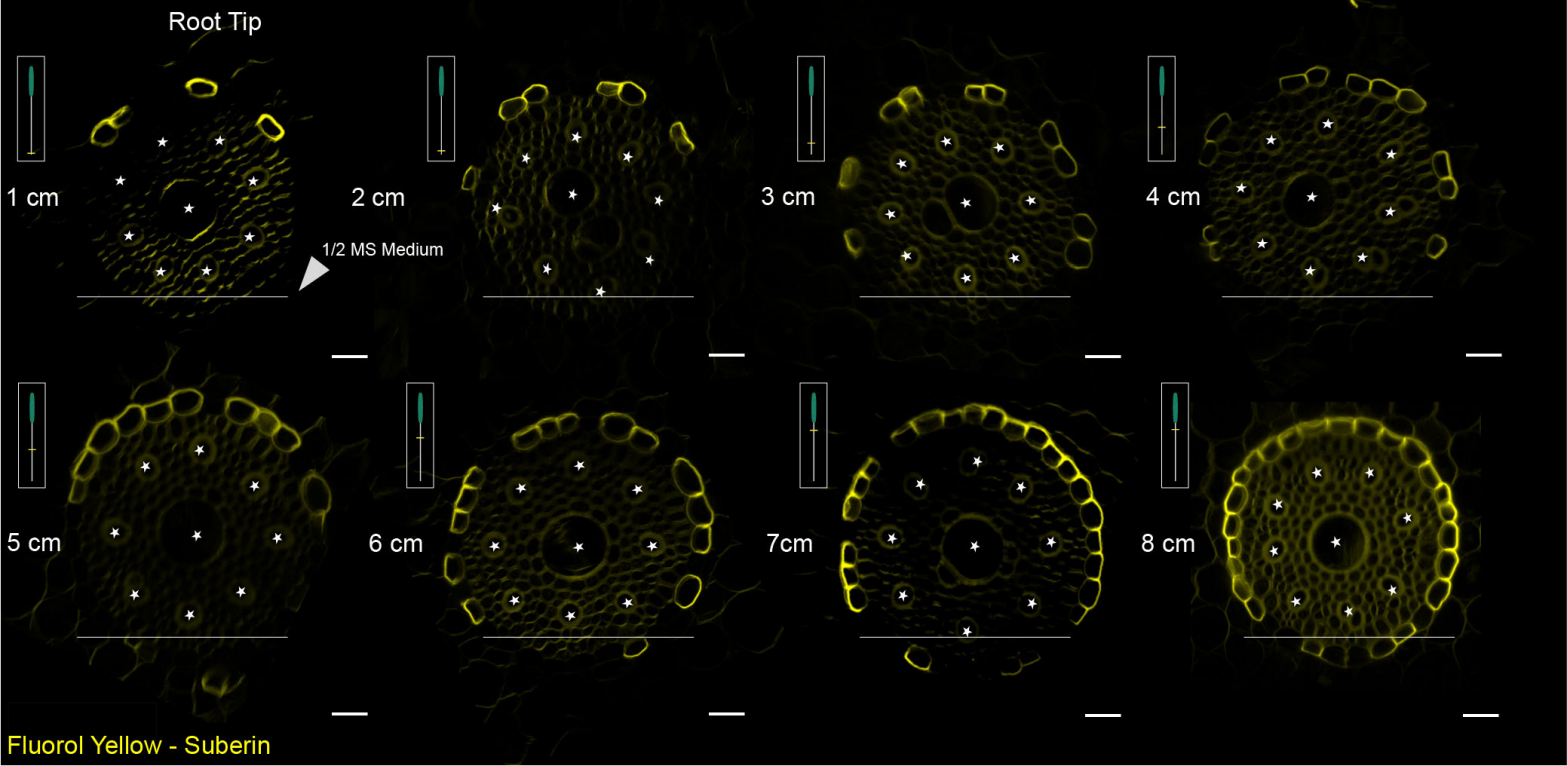
**Pattern of endodermal suberization along the Brachypodium root axis adjacent to the nutrient medium growth.** Cross-sections of Brachypodium primary root showing asymmetric suberization. SL developed unilaterally on the side of the root exposed to the air from the root apex, but not on the side exposed to nutrient medium. Representative images were obtained from 30 seedlings of Bd21-3 from three independent replicates, each consisting of at least ten plants. Roots of similar length were positioned in parallel for consistency, and regions of interest of approximately 1 cm were sectioned. Scale bars: 20 μm.

During the growth of Brachypodium seedlings on plate, we observed a very strong hydropatterning effect (Fig. S3) (Orosa-Puente et al., 2018). Basically, all LRs emerged on the side of the root in contact with the growth medium. It is proposed that hydropatterning serves to ensure roots have access with to water and nutrients (Möller et al., 2017). Brachypodium, like many monocots, has an additional cell layer that undergoes localized suberin deposition, the exodermis (Sexauer et al., 2021). Using Fluorol Yellow (FY) staining of cleared roots, we confirmed that the pattern of endodermal and exodermal suberization in Brachypodium also occurs after CS establishment initially with patchy zones for both the endodermis and exodermis (Fig. 3, Fig. S7B). This observation is consistent with the findings reported in rice, barley and tomato (Cai et al., 2011; Líška et al., 2016; Cantó-Pastor et al., 2024). Interestingly, suberization in the exodermis appeared delayed compared to suberin deposition in the endodermis (Fig. S7B). As previously reported in maize, Brachypodium, when grown vertically on agar plates, shows that endodermis and exodermis cells closest to the growth medium are the last to deposit suberin (Fig. 3).

### Dividing endodermal cells overlying the LRP do not appear to establish a Casparian strip domain

**Fig. 4.**
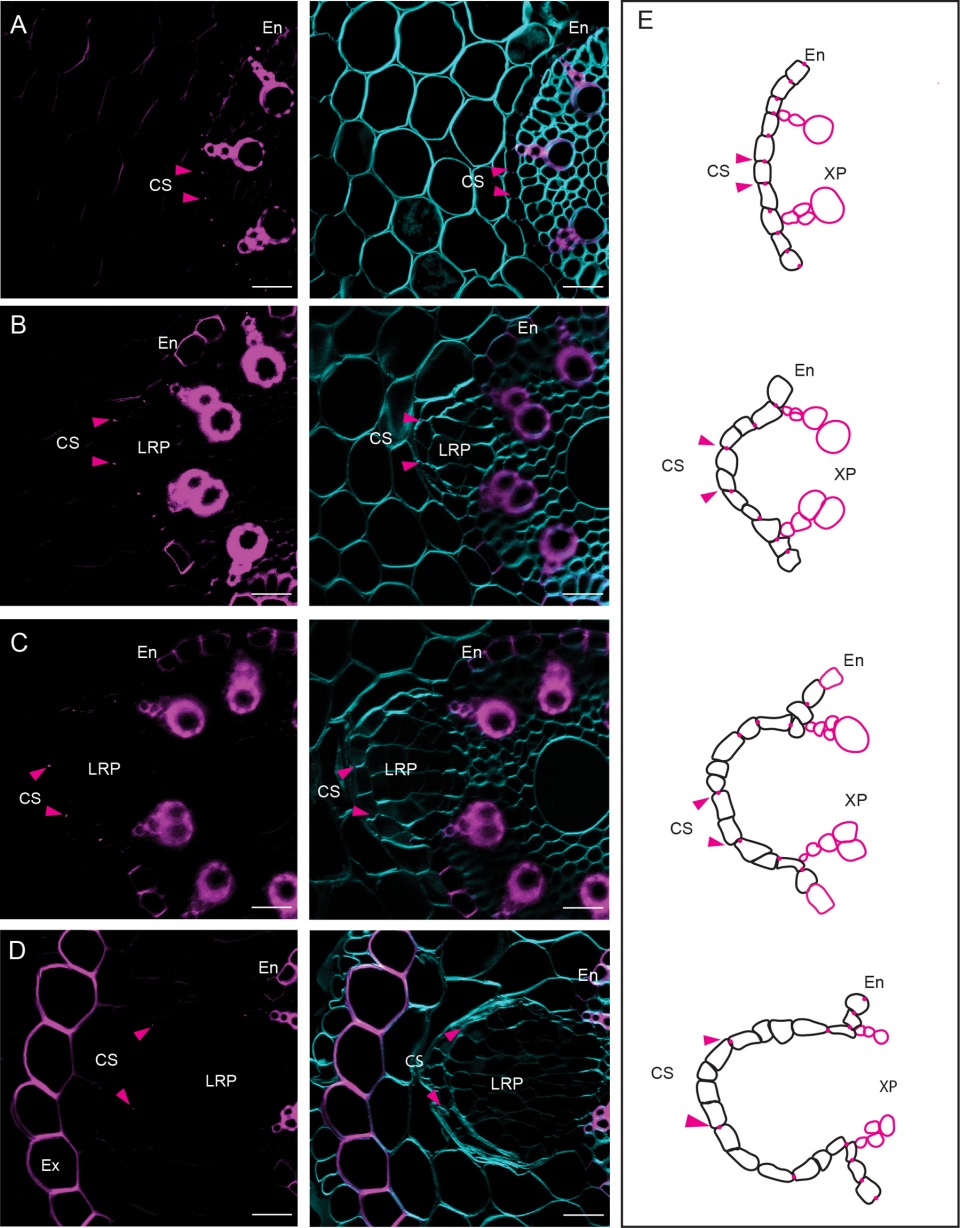
**Recently divided endodermal cells do not establish Casparian strips.** (A-D) Cross-sections stained with BF (lignin) in magenta and Renaissance SR2200 (cellulose) in cyan. The images illustrate the progression of cell divisions in the endodermis and the distancing of the previously formed CS. (E) A graphical representation depicting the cell divisions in the endodermis and the separation of the CS is shown on the right. The arrows indicate the position of the CS. Roots of seedlings (six DAG) with similar length were positioned in parallel for consistency, and regions of interest of approximately 1 cm from the root tip were sectioned. Representative images were obtained from 30 seedlings of Bd21-3 from three independent replicates, each consisting of at least ten plants. Scale bars: 50 µm.

The participation of the endodermis during LR organogenesis in Brachypodium is not unique, as it has been demonstrated already for a range of plant species (Xiao et al., 2019). Although we could not observe suberin deposition in the endodermis overlying the LRP under our growth conditions, the Casparian strip domain (CSD) and CS are already present in the overlying endodermis prior to LR initiation. However, little is known regarding the cell fate of the endodermis cells that reactivate their cell cycle and eventually become a part of the LRP. Do these cells after division establish a CSD that is attached to the CS? To address this, we used a histochemical staining for lignin (Basic Fuchsin) and cellulose (Calcofluor White) to counterstain cell walls. In root sections containing LRP, we observed that endodermal cells that underwent anticlinal divisions appear not to establish a CSD, similar to endodermal cells undergoing periclinal divisions, based on the absence of the characteristic lignified spot in the endodermal cross wall (Roppolo et al., 2011) (Fig. 4). From surface projections of root sections containing LRP in which the endodermis already underwent a few rounds of divisions, it appeared that no newly established CS were present in these endodermis cells (Fig. 4B-D). We observed that the CS appeared to undergo a regulated breaking like what was observed during Arabidopsis LR formation (Vermeer et al., 2014). In addition, we observed that the CS appears to undergo a lateral detachment (‘sliding’) to facilitate the outgrowth of the LRP (Fig. S8).

## DISCUSSION

**Fig. 5.**
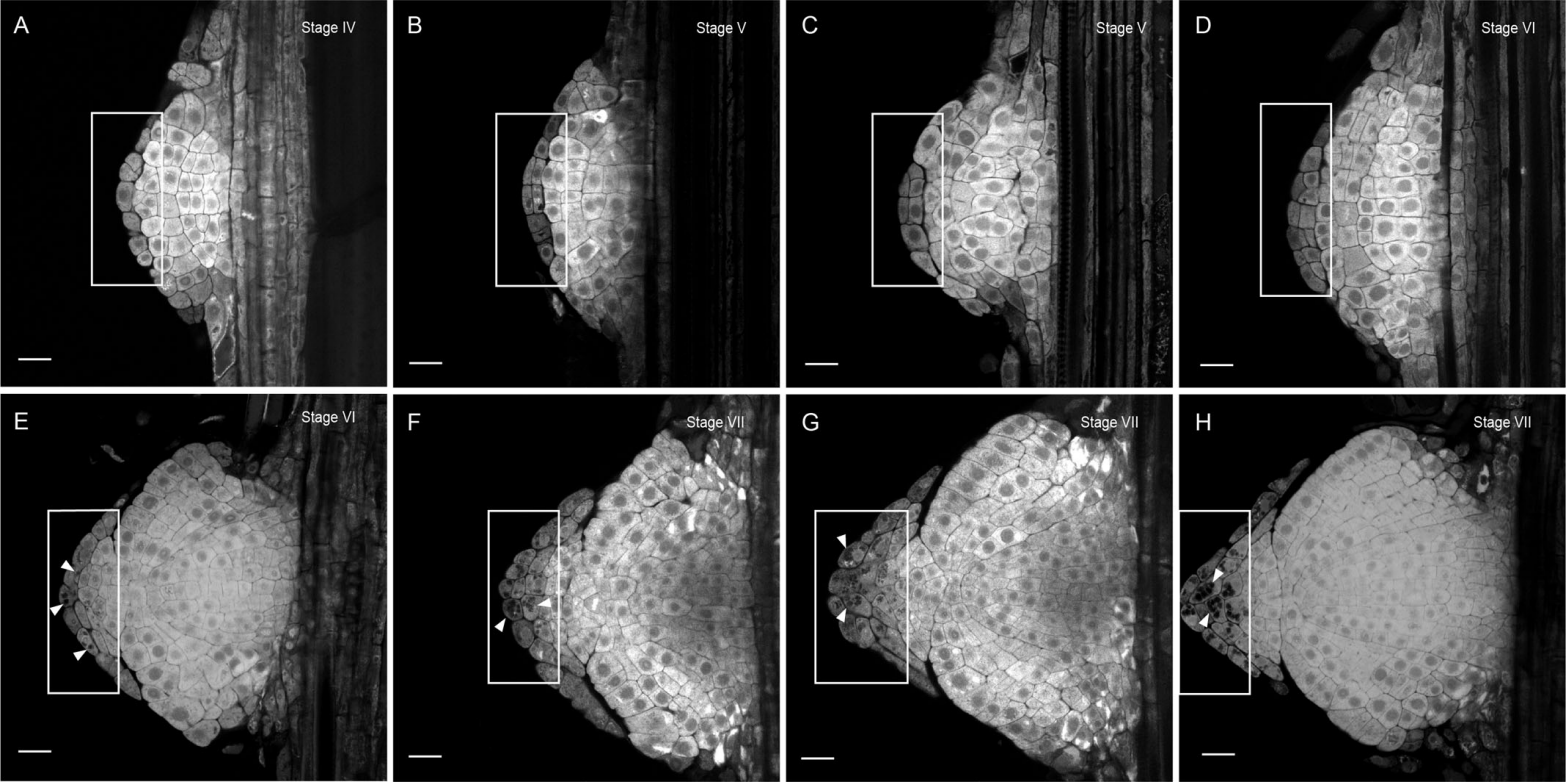
**Endodermal cells give rise to the columella cells of the root cap.** (A-H) Starch granules (dark structures in boxed area) were detected using Lugol staining. The boxed area shows cell divisions in the endodermis and its progression in differentiating into columella cells from Stage VI (E) to Stage VII (H) marked by the sediments of starch granules (arrowheads) in the first cell layer on the apex of a Stage VI LRP. Representative images were obtained from 30 seedlings from three independent replicates, each consisting of at least ten plants of Bd21-3. The root cortex was mechanically removed with forceps preserving the LRPs integrity. Samples were cleared with DEEP-Clear and stained with Lugol. Scale bars: 50 µm.

In this study, we present an atlas describing the consecutive stages of LR development in Brachypodium based on the model utilized for Arabidopsis (Malamy and Benfey, 1997; Péret et al., 2009; Van Norman et al., 2013; Vermeer and Geldner, 2015; Wachsman and Benfey, 2020). We show that in Brachypodium, like other monocots, pericycle cells adjacent to phloem are competent for LR organogenesis. However, to really map the origin and contribution of pericycle cells, a clonal analysis would be required. This approach was recently used to show that also phloem pole pericycle cells can contribute to LRP in Arabidopsis (Torres-Martínez et al., 2020). Thus, it would be interesting to use clonal analysis to determine whether the xylem pole pericycle (XPP) could contribute to LR formation in Brachypodium, especially during later developmental stages. Furthermore, the endodermis undergoes mitotic activation soon after the initial pericycle cell divisions and the overlying endodermal cells will become an integral part of the LRP, as they will form the root cap (Figs 1 and 5). While the inner-most cortex layer appears to undergo cell divisions, we could not confirm whether these cells become part of the LRP itself, like the overlying endodermal cells. In both cases, usage of clonal analysis would be very useful to trace the cell fate of the endodermis-derived cells during LR development. Alternatively, we hypothesize these divisions are required to accommodate the expansion growth of the LRP, thereby facilitating emergence. Although most of textbook knowledge regarding LR development is based on Arabidopsis studies, the mitotic reactivation and participation of the endodermis and derived cells are observed in a large number of plants species including barrel clover (Herrbach et al., 2014), maize (Jansen et al., 2013) barley (Orman-Ligeza et al., 2013) and many others (Xiao et al., 2019). It appears that absence of the incorporation of the endodermis during LRP growth could be specific for the Brassicaceae (Xiao et al., 2019). Alternatively, we hypothesize that these divisions are necessary to accommodate the expansion growth of the LRP, potentially aiding in its emergence by facilitating growth through the overlying both the endodermis and adjacent cortex cell layers (Bell and McCully, 1970; Torres-Martínez et al., 2020). The mitotic reactivation of the endodermis raises important questions: What triggers this process and do the dividing endodermal cell change their identity and if so at what stage? At which stage do they obtain columella identity? To address these questions follow-up studies employing sectorial (mosaic) analyses and high-resolution expression analysis (single cell/nuclei sequencing, spatial transcriptomics) (Birnbaum, 2018; Torres-Martínez et al., 2020; Liu et al., 2023) would be a logical step to track cell lineages and changes of cell identity during the LRP developmental process.

Auxin serves as a crucial regulator of LR patterning, development and the DR5 reporter is commonly employed to visualize auxin responses (Ulmasov et al., 1997; Liao et al., 2015). In this study, we could not detect the DR5pro::ER-mRFP (DR5) signal in phloem pole pericycle cells during the formative cell divisions leading to a stage I LRP. However, there was induction of DR5 signal in the overlying endodermis and even more so in the cortex cells overlying the LRP. Interestingly, we also observed during later stages of LRP development, clear induction of the DR5 signal in the exodermis overlying the LRP. This suggests for a similar role of auxin signaling to regulate cell wall modifications to facilitate the emergence of the LRP (Swarup et al., 2008; Meng et al., 2019). The observed induction of the DR5 reporter in the overlying exodermis suggest for a possible role for auxin signaling to regulate cellular responses, such as modification of the lignin barrier, to accommodate emergence (Nakayama et al., 2017).

Previous studies have reported the absence or presence of a weak DR5 signal during the first cell divisions in the pericycle of rice (Ni et al., 2014) barley (Kirschner et al., 2017) and maize (Jansen et al., 2012) the opposite of what is commonly observed in Arabidopsis (Dubrovsky et al., 2000; Vanneste et al., 2005; Marhavý et al., 2013). Similarly, during many stages of Brachypodium embryo development, the DR5 signal was not or barely detected (Hao et al., 2021) counterintuitive compared to the observations during Arabidopsis embryogenesis (Möller and Weijers, 2009). The synthetic DR5 promoter contains direct repeats of a medium-affinity biding site for the AUXIN RESPONSE FACTORs (ARF) transcriptional regulators (Ulmasov et al., 1997; Boer et al., 2014). Therefore, it is likely that only part of the transcriptional response to auxin is reported. The use of higher affinity binding sites could provide a solution to better address the role of auxin during early developmental processes in Brachypodium (Liao et al., 2015; Hao et al., 2021). The same DR5 reporter has been used to monitor changes in auxin signaling during Brachypodium spikelet formation in the shoot (O'Connor et al., 2017). In addition, we observed a clear DR5 signal during later stages of LRP development, including in the cortex and exodermis. A plausible explanation could be that a set of ARFs with reduced affinity for the DR5 promoter are regulating auxin responses during early stages of LR development in Brachypodium. It would also be important to test brighter and/or triple-fluorescent protein fusions that are targeted to the nucleus, or radiometric reporters such as R2D2 to might be better suited at reporting auxin signaling (Liao et al., 2015). It is clear that auxin can induce LR formation in Brachypodium (Pacheco-Villalobos et al., 2013) and important regulators of auxin import and efflux are already expressed in stage I LRP (Fig. S5).

The root endodermis serves as apoplastic barrier for the radial transport of water and nutrients to the plant's vascular system (Barberon et al., 2016). To fulfil this role, the endodermis relies on the formation of the lignified Casparian strips. Subsequently, suberin lamellae are deposited as a secondary cell wall modification surrounding the plasma membrane (Barbosa et al., 2019). Moreover, many plant species have an extra barrier called the exodermis, which also exhibits lignin and suberin deposition (Kajala et al., 2021; Liu and Kreszies, 2023). Recent studies have reported that the exodermis functions in the tolerance to abiotic stresses (Cai et al., 2011; Kajala et al., 2021; Manzano et al., 2022 preprint; Cantó-Pastor et al., 2024). Here, we demonstrate that the daughter cells of divided endodermal cells do not appear to form a CSD in their cross walls (Fig. 4). Similar to what was described for Arabidopsis, the CS appears to be detached longitudinally and local breaks appear, likely facilitating the emergence of the LRP. While there is increasing interest in studying the function and formation of the CS in monocots such as rice and maize (Karahara et al., 2004; Wang et al., 2022), little is known whether the CASPARIAN STRIP DOMAIN PROTEINs (CASP) are degraded during LR development as described for Arabidopsis (Vermeer et al., 2014) to facilitate the reported sliding of the CS. In addition, it would be interesting to test whether Brachypodium has orthologs of the GAPLESS proteins that were identified in rice. These secreted proteins interact with OsCASP1 and are required for the tethering of the CS to the cell wall (Song et al., 2023). It will be interesting to test whether CASP/CSD degradation could be a general mechanism to break the GAPLESS-mediated tethering of the CS to the cell wall to allows for loosening and/or local breaking of the CS in plants during LR emergence. Moreover, Brachypodium has a lignified exodermis (Sexauer et al., 2021). However, under our experimental conditions, the exodermis in many cases showed still little lignification at the time of LR emergence.

## Concluding remarks

Here we provide an atlas describing the various developmental stages of LRP development in Brachypodium and shed light on the potential roles of different cell types and molecular mechanisms involved to facilitate their development. This now provides a perfect starting point to dissect the trajectories of cell types and if there are regulatory mechanisms that could be part of conserved modules for root branching in general. Brachypodium LRP formation provides a beautiful (non-domesticated) plant model to investigate how the endodermis and cortex re-activate their cell cycle and contribute to organogenesis and emergence. In addition, it allows the investigation as to which (hormonal) signaling pathways are re-wired and which are conserved during developmental processes.

## MATERIALS AND METHODS

### Plant materials and growth conditions

*Brachypodium distachyon* seedlings (Bd-21-3) (Vogel and Hill, 2008) were grown vertically on 0.8% agar supplemented with half strength Murashige-Skoog (MS) pH 5.8 at 22°C under long day or constant light. Five DAG seedlings were collected for analysis. After removal of the seed husk, seeds were surface sterilized using sodium hypochlorite 5% and 0.01% Triton for 4 min and rinsed at least four times in autoclaved desilted water. Seeds were placed on medium (prepared as described above) with embryo towards the bottom of the and facing the lid of the 120 mm square plate to prevent shoots and roots growing into the media or in the wrong direction. Plates were placed into growth conditions at an angle of about 20° to ensure that roots grow on the medium and not into the air as described previously (van der Schuren et al., 2018). After a maximum of 7 days in 22°C under long day or constant light, seedlings were harvested for clearing and analysis. The description of the Brachypodium LR developmental stages was based on representative images obtained from 45 seedlings from three independent replicates each consisting of at least 15 plants.

### Auxin treatment

A total of 45 seedlings (three independent biological replicates) of Brachypodium Bd-21-3 were grown vertically under long day conditions for 5 days on standard ½ MS plates with 0.8% agar. The seedlings were then transferred to plates treated with 10 µM Indole-3-Acetic Acid (IAA) (I0901, Duchefa Biochemie) from a 10 mM stock in DMSO. Images were taken at 0, 3, and 7 days after the seedlings were transferred to auxin.

### Chemicals for clearing and staining solutions

The following chemicals were used in the DEEP-Clear (Pende et al., 2020) adapted version to plant tissues: PFA (paraformaldehyde) (CAS no. 30525-89-4, Merck, http://www.merck.com/), xylitol (CAS no. 87-99-0, Sigma-Aldrich, http://www.sigmaaldrich.com/), urea (CAS no. 57-13-6, Sigma), SR2200 (Renaissance Chemicals), Basic Fuchsin (CAS no. 58969-01-0, Sigma-Aldrich), THEED (Sigma-Aldrich, 87600-100ML), 5% (v/v) Triton X-100 (Roth, 3051.2).

### Preparation of hand-sectioned root samples

For sectioning, seedlings roots (six DAG) of similar length were placed in parallel and fragments of 1 cm with the region of interest were partitioned and embedded in 4% agarose. After solidified, agarose blocks containing the region of interest were glued on a hand microtome (www.daigger.com/hand-microtome) and sections of approximately 50 μm were prepared for clearing or immediate visualization. Representative images were obtained from at least 30 seedlings from three independent replicates.

### Clearing and staining

Clearing steps using DEEP-Clear were performed as described for ClearSee in (Kurihara et al., 2015) and adapted from (van der Schurenet al., 2018) for *Brachypodium* samples. DEEP-clear solution consists in 5 to 8% (v/v) THEED, 5% (v/v) Triton X-100, and 25% (w/v) urea in water. Heating the solution is not recommended. Seven DAG old root seedlings were collected for clearing for full root treatment and/or for semi-thin sectioning. Samples were fixed for 1 h in 4% (w/v) paraformaldehyde in 1× phosphate-buffered saline (PBS) with three rounds of soft vacuum infiltration. After, roots were washed five times in 1× PBS with another round vacuum to ensure the removal of PFA. Samples were then transferred to DEEP-Clear solution for clearing. Fixed root tissue was incubated at room temperature with gentle shaking for 7-10 days and solution replaced twice. For staining of the fixed and cleared tissue, 1% stock solution of Basic Fuchsin (for lignin staining), FY (for suberin staining), Renaissance and/or Calcofluor (for cell wall staining) were separately prepared directly in DEEP-Clear and stored at 4°C. Working solutions were prepared as in (Ursache et al., 2018). In order to combine multiple dyes, samples were incubated first in Basic F (0.1% in DEEP-Clear) for 1 h and washed in DEEP-Clear overnight. After, several rounds of washings, samples were transferred to Renaissance (0.1% in DEEP-Clear) for 2 days and washed overnight in DEEP-Clear. Finally, samples were transferred to FY (0,01% in DEEP-Clear) for 1 h and counterstained in Aniline Blue (0.5% in water) for 1 h in the darkness. Samples prior FY staining can be stored in 50% glycerol at 4%. FY solutions and FY-stained samples were kept in darkness to prevent bleaching.

### Starch staining

To observe starch granules in the LRPs of Brachypodium, the root cortex of a total of 30 seedlings (three independent biological replicates) was mechanically removed without damaging the LRPs. Roots were then cleared for 3 days in DEEP-Clear. After, roots were dipped in Lugol's staining solution (Sigma-Aldrich) for 5 min, washed with distilled water, and observed under 2-photon microscopy.

### Microscopy

Roots were observed using Leica TCS SP8-MP equipped with a resonant scanner (8 kHz) using 25×, 40× and 63× water immersion objectives. Figures were arranged in Adobe Illustrator (Adobe Systems Inc., http://www.adobe.com/) or in PowerPoint (Microsoft Corporation) and the brightness was increased equally, without further modifications. The 3D reconstruction was done using the Fiji package (Schindelin et al., 2012).

## Supplementary Material

10.1242/biolopen.060531_sup1 Supplementary information
