# Supplementary material for: An atlas of Brachypodium distachyon lateral root development
Source: Biol Open. 2024 Sep 2;13(9):bio060531. doi: 10.1242/bio.060531 (PMC11391822; doi:10.1242/bio.060531)
Supplement: Supplementary information [file biolopen-13-060531-s1.pdf]

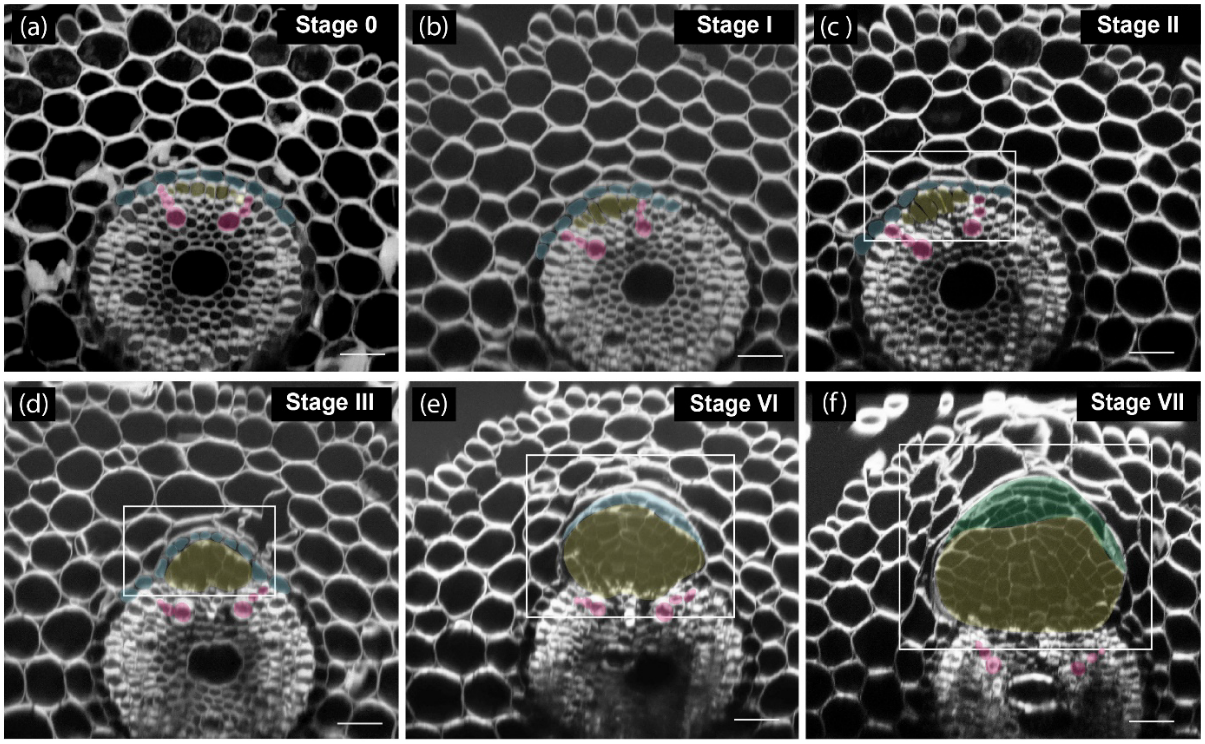

Fig. S1. Orthogonal view of developmental stages of LRP formation in Brachypodium. (**A**) Stage **0**: No evident swelling of the pericycle cells (white arrows). (**B**) Stage **II**: Recently divided pericycle cells promote the displacement of the endodermis. In (**C** and **D**) Stage **II** and **III** pericycle divides anticlinal and periclinal (rectangular area). (**E** and **F**) - Periclinal and anticlinal cell divisions are observed at the apex of the LRP (Stage **V**) following the establishment of the lateral root cap (Stage **VII**). Representative images were obtained from 30 seedlings from three independent replicates, each consisting of at least 10 plants. Magenta: Xylem, Cyan: Endodermis, Yellow: Pericycle, Green: Root Cap. Scale: 50 μm.

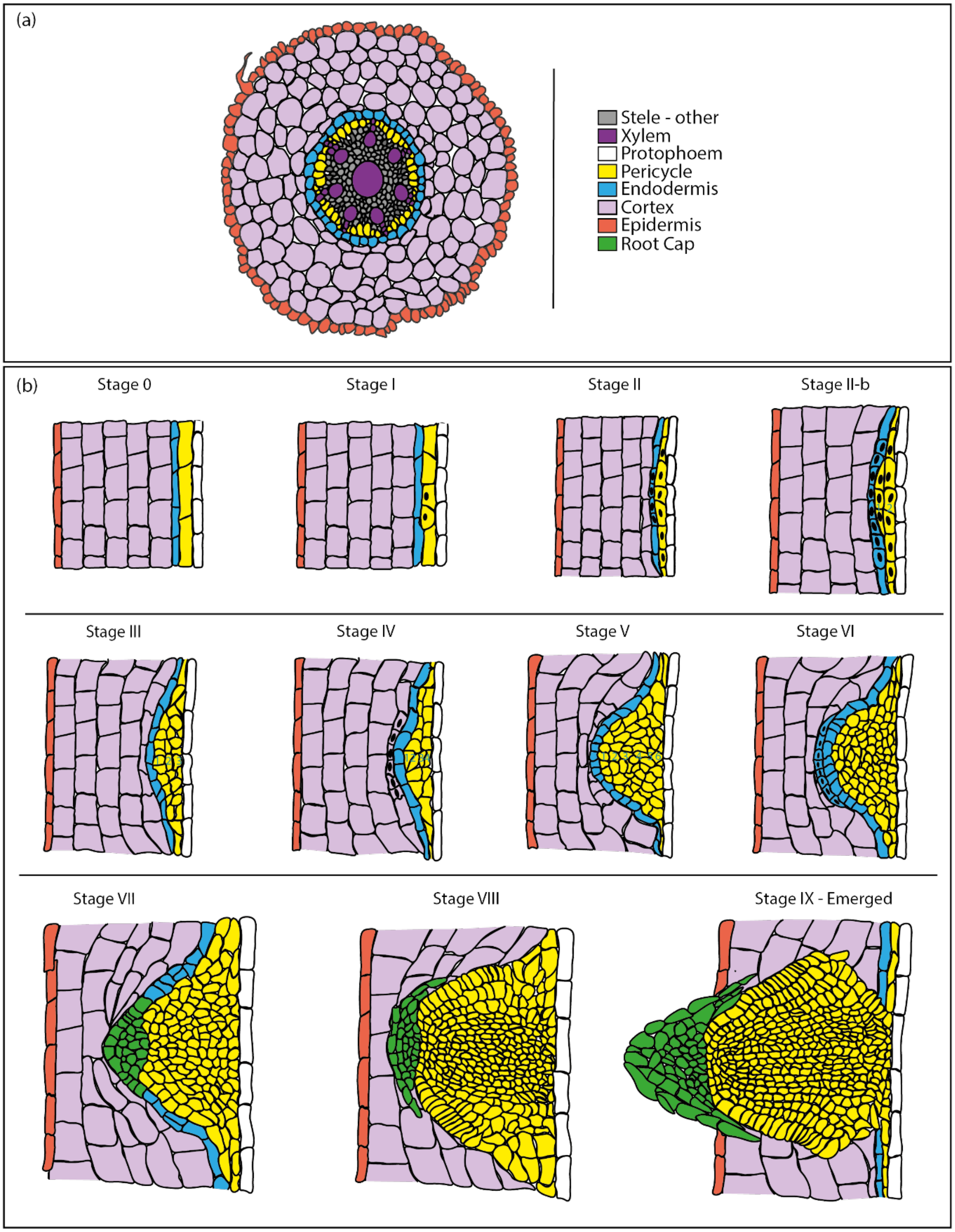

Fig. S2. Schematic representation of LRP development in Brachypodium. (**A**) Representation of root cross section of Brachypodium. (**B**) Successive stages of LRP formation are illustrated.

Biology Open • Supplementary information

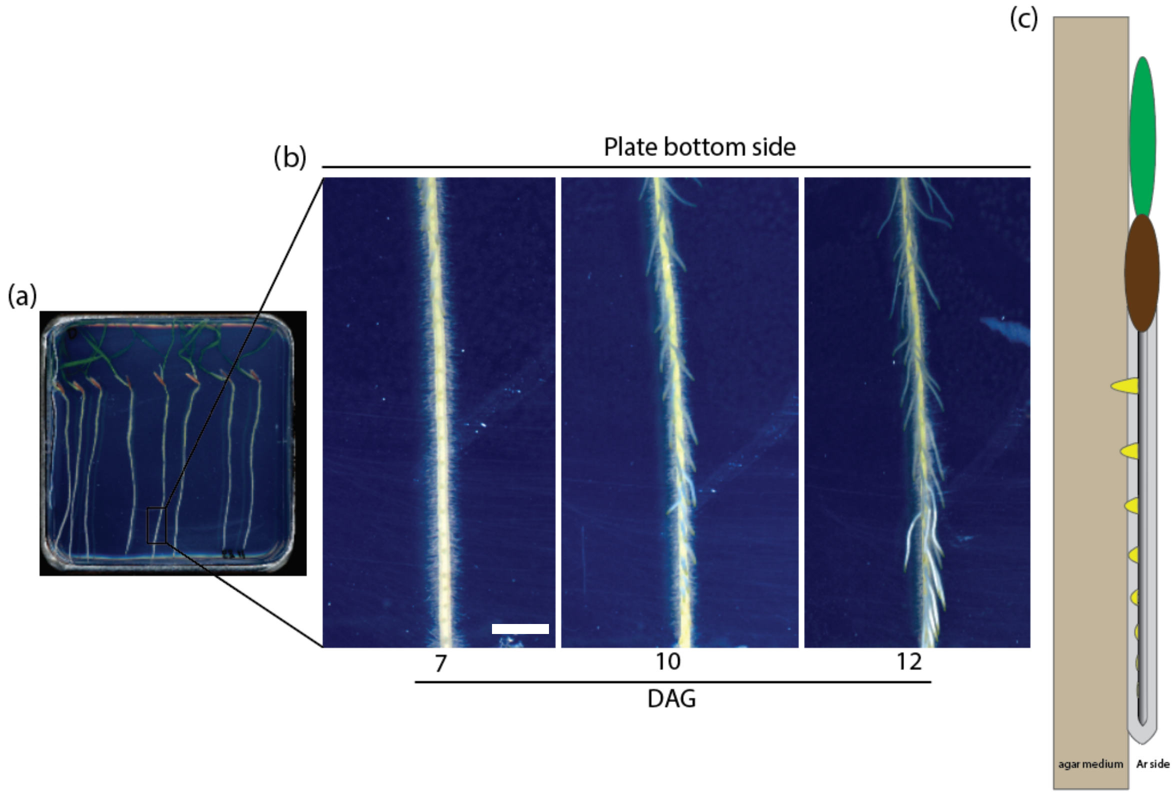

Fig. S3. Brachypodium LRs emerge towards the agar medium.

(**A**) Brachypodium seedlings grown on 12x12 cm plates supplemented with half-strength MS solution. (**B**) Progression of lateral root emergence towards the agar after 7, 10, and 12 days after germination (DAG). (**C**) Side view illustration of lateral roots growing towards the agar medium. Representative images were obtained from 45 seedlings from three independent replicates, each consisting of at least 15 plants. Scale: (**B**) 3 mm.

Biology Open • Supplementary information

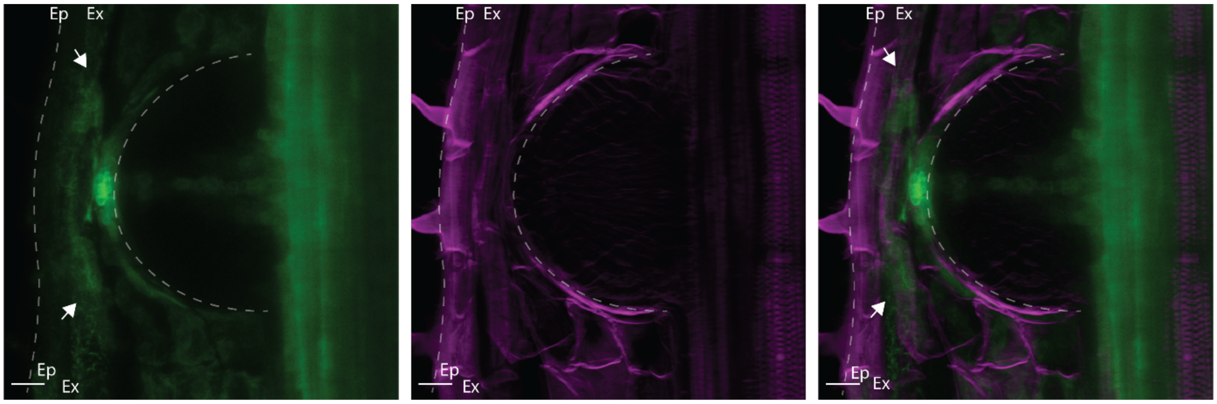

Fig. S4. ***DR5pro::ER-mRFP*** **is induced in the apex of the LRP and in the overlying exodermis.** The white arrow indicates presence of the DR5 signal (green) confined to the overlying exodermis. Representative images were obtained from 15 seedlings from three independent replicates, each consisting of at least 5 plants. *DR5pro::ER-mRFP* (green) and cells walls stained with SCRI Renaissance (magenta) for cellulose. Ep: Epidermis, Ex: Exodermis. Scale bar = 20 μm.

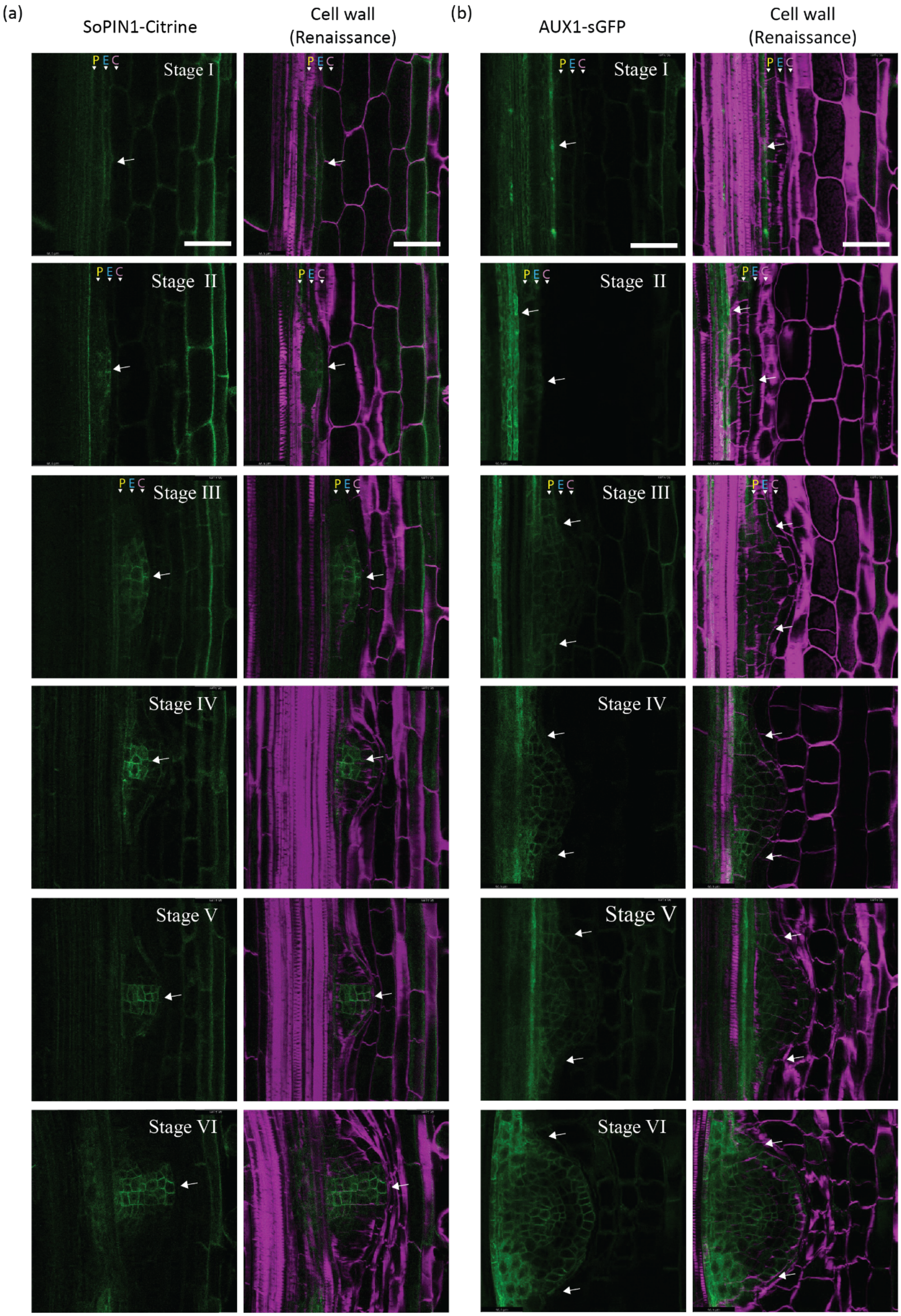
(a)
SoPIN1-Citrine
Cell wall (Renaissance)
(b)
AUX1-sGFP
Cell wall (Renaissance)
P E C
Stage I
Stage II
Stage III
Stage IV
Stage V
Stage VI

Fig. S5. Localization of *SoPIN1-Citrine* and *BdAUX1-sGFP* during LR formation in Brachypodium.

(**A**) SoPIN1-Citrine is observed from Stage I (white arrowhead) during the initial cell divisions in the endodermis. Later, expression is localized in the central region of the LRP. (**B**) Expression of AUX1-sGFP is observed in the vasculature of the primary root and in the LRP from Stage I (white arrowheads). During later stages of LR development, AUX1-sGFP signal is observed in the flanking regions of the LRP with its intensity increasing subsequently in both the vasculature and endodermis in Stage VI. Representative images were obtained from 30 seedlings from three independent replicates, each consisting of at least 10 plants. SoPIN1-Citrine and BdAUX1-sGFP (green) and cells walls stained with SCRI Renaissance (magenta) for cellulose. Scale bar = 50 μm.

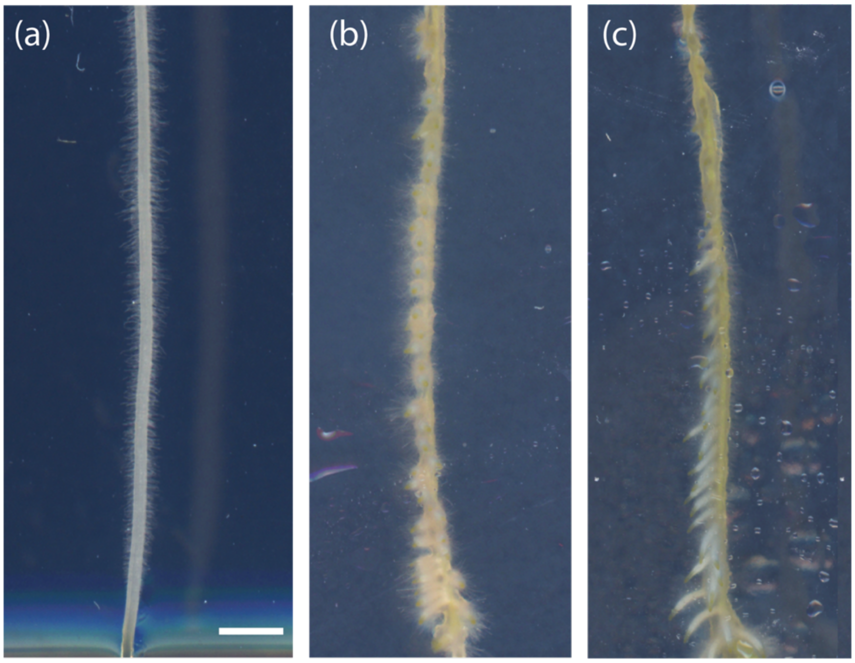

Fig. S6. Exogenous auxin addition induces lateral root formation in Brachypodium. Seedlings were grown on standard ½ MS plates for 6 days and transferred to auxin treatment (10 µM IAA) for 0 (**A**), 3 (**B**), and 7 days (**C**). Scale bar: 0.3 cm. Representative images were obtained from 45 seedlings from three independent replicates, each consisting of at least 15 plants of Bd21-3. Scale bar = 3 mm.

Biology Open • Supplementary information

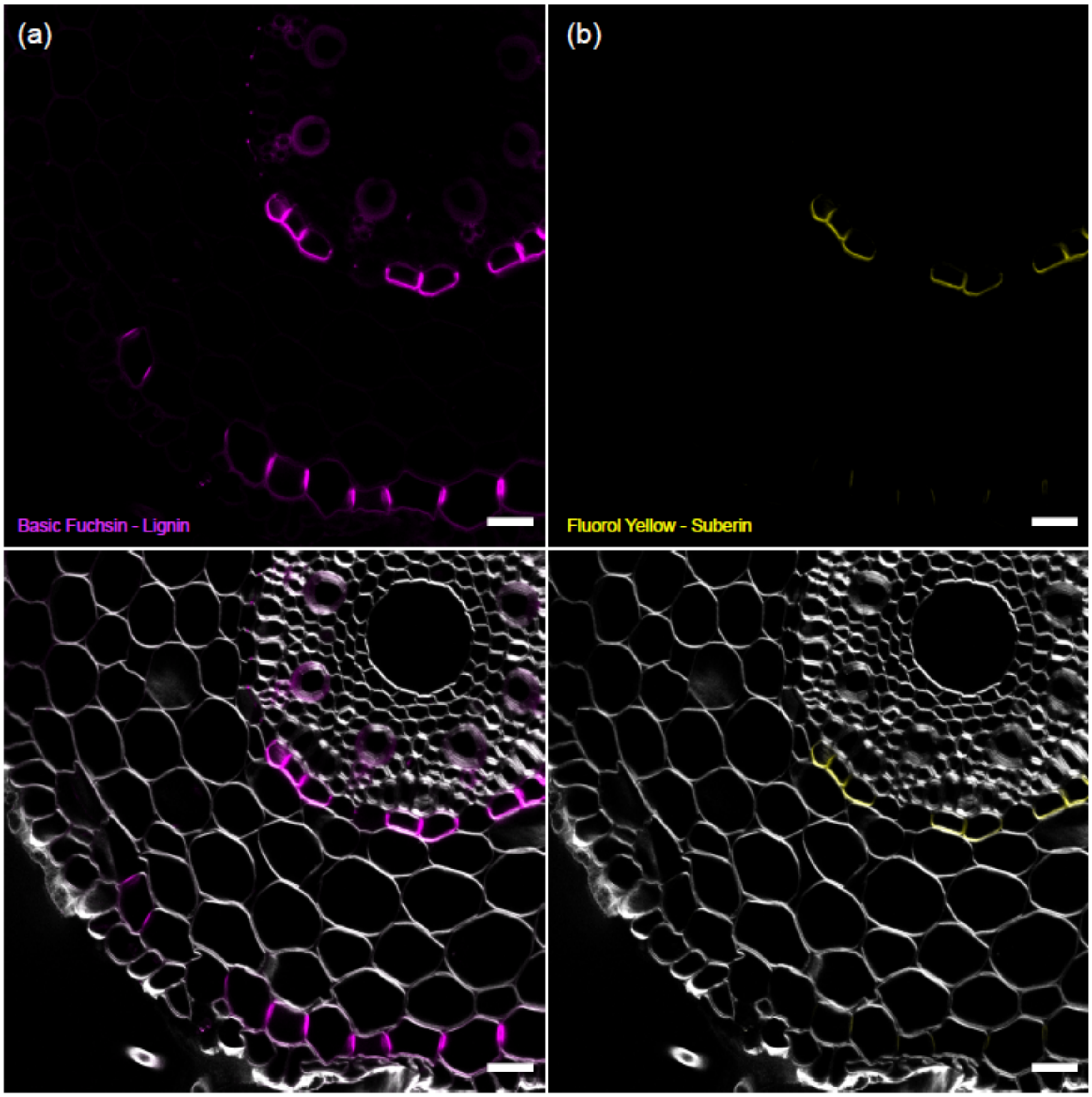

Fig. S7. The exodermis shows delayed suberization compared to the endodermis. Cross-sections of Brachypodium primary roots double stained for (**A**) lignin (BF) (**B**) suberin (FY) counter stained with Renaissance SR2200 (cellulose). Roots of seedlings (6 DAG) with similar length were positioned in parallel for consistency, and regions of interest of approximately 1 cm from the root tip were sectioned. Representative images were obtained from 30 seedlings of Bd21-3 from three independent replicates, each consisting of at least 10 plants. Magenta = BF/lignin, Yellow = FY/suberin and gray = SR2200/cellulose. Scale bar: 50 µm.

Biology Open • Supplementary information

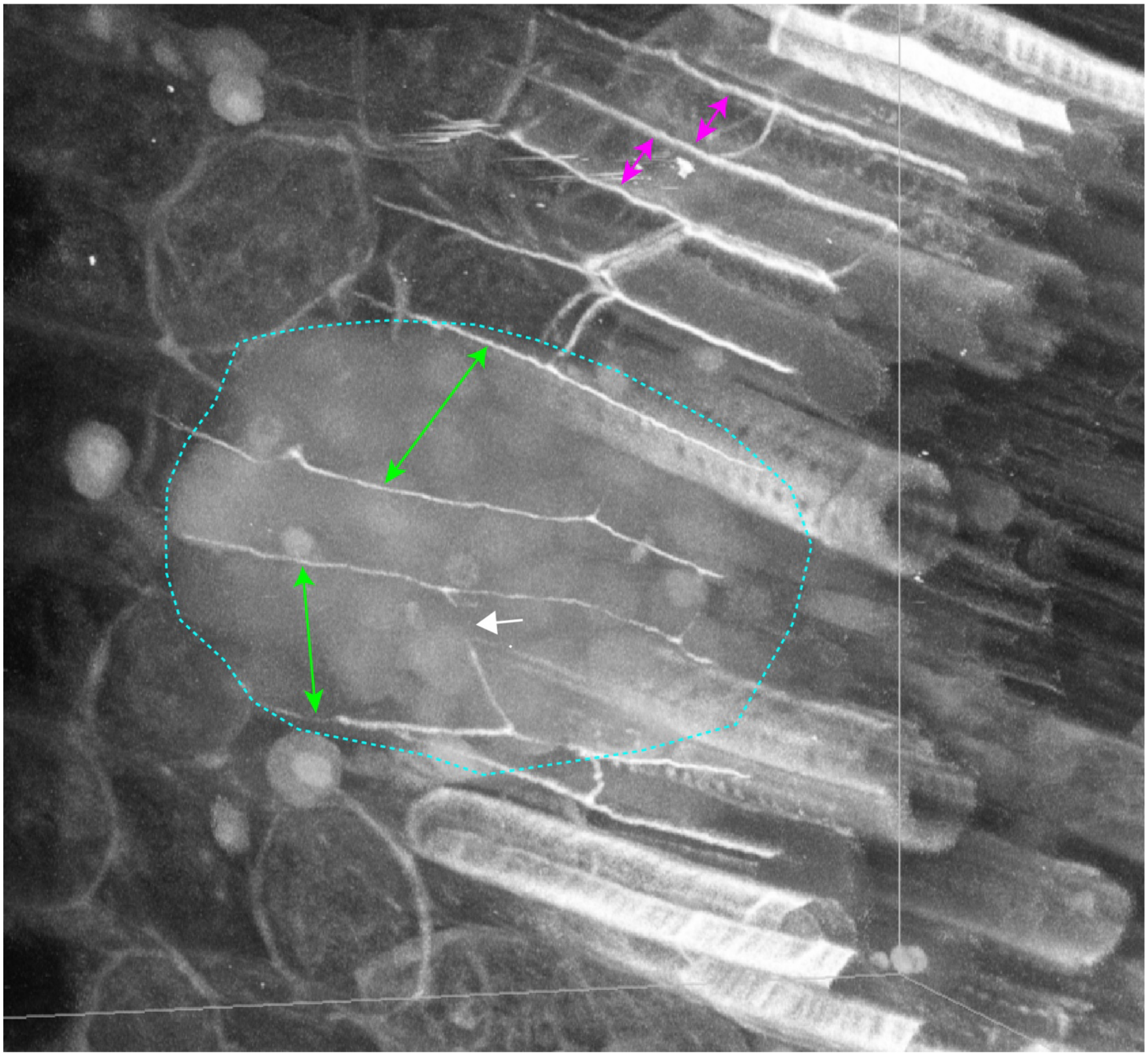

Fig. S8. The CS undergoes lateral detachment during LRP development in Brachypodium. Maximum image projection of a cleared root stained with Basic Fuchsin. The CS breaks locally (white arrow) and appears to be laterally displaced (compare spacing between CS indicated overlying a LRP (green double arrow) and without the presence of a LRP (magenta double arrow) during the emergence of the LRP, outlined by dotted cyan line. Representative images were obtained from 15 seedlings of Bd21-3 from three independent replicates, each consisting of at least 5 plants. BF/lignin = gray. Scale bars = 20 µm.
